# Antipsychotic prescribing practices and their association with rehospitalization in a forensic psychiatric sample

**DOI:** 10.3389/fpsyt.2024.1474626

**Published:** 2024-10-25

**Authors:** Joseph Goody, Karen Petersen, Johann Brink, Anne G. Crocker, Tonia Nicholls

**Affiliations:** ^1^ Faculty of Medicine, Department of Psychiatry, University of British Columbia, Vancouver, BC, Canada; ^2^ Department of Psychiatry and Addictions, School of Criminology, Université de Montréal, Montréal, QC, Canada; ^3^ Institut national de psychiatrie légale Philippe-Pinel, Montreal, QC, Canada; ^4^ Department of Psychiatry, University of British Columbia, Vancouver, BC, Canada

**Keywords:** antipsychotic, prescribing practices, forensic, psychiatry, rehospitalization, clozapine

## Abstract

While there is extensive literature examining the effectiveness of antipsychotic prescribing to patients with schizophrenia spectrum or other psychotic disorders in general psychiatric services, there is a dearth of studies examining antipsychotic prescribing practices and their effectiveness in forensic psychiatric services. Forensic psychiatric patients have unique challenges often due to their high-profile offences, public scrutiny, and legal requirements. This longitudinal, retrospective study aimed to examine antipsychotic prescribing and rehospitalization rates in a forensic psychiatric sample, along with relevant socio-demographic, clinical, and forensic characteristics. All patients had a psychotic illness and were prescribed antipsychotic medication. The sample included 153 patients, of which the majority were male (85.6%), Caucasian (71.2%), middle aged (30s to 50s), had schizophrenia or schizoaffective disorder (76.5%), had a substance use disorder (62.1%), and had a most serious index offence against the person (80.4%). Atypical antipsychotics accounted for the majority of antipsychotic prescriptions (75.9%) and the sample had an antipsychotic polypharmacy rate of 39.9%. The sample was divided into four primary antipsychotic formulation types, which were oral (34.0%), injection (39.2%), clozapine (19.0%), and subtherapeutic (7.8%). Regarding rehospitalization, 52.9% of the sample was rehospitalized, with the average number of rehospitalizations being 1.2 (*SD* = 1.7) and proportion of the follow up period rehospitalized being 16.4% (*SD* = 27.7%). Patients prescribed clozapine had numerically lower rates of rehospitalization than those prescribed oral and injection formulation types, but it was not statistically significant. With a 19.0% prescription rate, clozapine may be underutilized in this sample. Further research is needed to demonstrate the potential benefits of clozapine regarding rehospitalization in forensic psychiatric patients, as has already been done in general psychiatry. Advancing treatment of the high-profile forensic population can reduce stigma toward people with mental illness and criminal justice involvement.

## Introduction

The vast majority of patients receiving forensic mental health services have schizophrenia spectrum disorders or other psychotic disorders (SSD) as their primary presenting mental health needs and are treated with antipsychotics (1–4). There are many guidelines available to advise physicians on the treatment of SSD, such as those from the Canadian (CPA) and American (APA) Psychiatric Associations and the National Institute for Health Care and Excellence (NICE) in the UK (5–7). However, these guidelines were designed for general psychiatry, and do not specifically comment on the forensic psychiatric population (5–7). The APA guidelines suggest that patients with schizophrenia and a high risk of aggressive behavior should be treated with clozapine, and patients with poor medication compliance be treated with a long-acting injectable antipsychotic (LAIA) (6). But the patients in those settings are also different from forensic patients in Canada who are, for instance, deemed by the courts not criminally responsible on account of mental disorder (NCRMD). In general, forensic patients are more likely to be treatment-resistant, violent, and to have complex comorbid problems than patients in general psychiatry (1, 4). This makes general guidelines less transferrable, and there is a paucity of specific literature available to inform forensic mental health clinicians on how to address the needs and preferences of this uniquely challenging population (4). With respect to populations with criminal justice involvement, the Canadian (CAPL) and American (AAPL) Academies of Psychiatry and the Law and European Psychiatric Association (EPA) produced guidelines for prescribing in correctional facilities, such as jails, or other forensic non-hospital settings (8–10). Consistent with the general psychiatric guidelines from CPA, APA, and NICE, the CAPL, AAPL, and EPA also suggest clozapine as an option to reduce the risk of violence in people with schizophrenia and LAIAs to improve medication adherence (8–10).

In non-treatment-resistant schizophrenia, guidelines recommend tailoring the choice of antipsychotic to the individual needs and preferences of each patient, considering efficacy and side effects (5–9). For treatment-resistant schizophrenia, clozapine is the first line agent (5–9). Recent meta-analyses and a meta-review have confirmed that clozapine is superior to other 1^st^ and 2^nd^ generation antipsychotics for treatment-resistant schizophrenia, with approximately 40% of clozapine-treated patients achieving a significant reduction in psychotic symptoms (11–13). Land et al. published a meta-analysis in 2017 which showed clozapine reduced the proportion of people hospitalized compared to all other studied antipsychotics, save haloperidol and depot medication (14). The meta-analysis included 37 studies from 1990 to 2016 conducted in Europe, USA, Canada, South America, and Asia; the sample size for clozapine was 12,631 and 35,337 for control medication (14). Clozapine achieved significance even over depot medication, when one outlying study comparing clozapine to perphenazine depot was removed (see Land et al., 2017; 14). However, evidence suggests that clozapine is often delayed or not used when indicated, despite its known benefits (5, 6, 11).

In many countries, clozapine is restricted to patients with treatment-resistant schizophrenia due to concern over its potentially fatal side effects, such as agranulocytosis, myocarditis/cardiomyopathy, and metabolic syndrome (15). However, in 2019, a meta-analysis showed that continuous clozapine users had significantly lower all cause mortality during a median of seven years follow up compared to other or no antipsychotics (15). Clozapine also appears to have an anti-aggressive effect which may be greater than its antipsychotic and sedative effects, and which is superior to other antipsychotics in treating aggression associated with psychosis (5, 16–18). This is particularly important in forensic populations, where clozapine offers the possibility of shorter custodial dispositions, reduced recidivism/return to custody, and better quality of life (17).

Along with clozapine, LAIAs have also been proposed as a preferred method for treating people with SSD in forensic settings (2, 5, 6). Some of the advantages of LAIAs include improved adherence and bioavailability and a more constant blood level (2). On the other hand, many patients have needle phobia, and “forcing” depot medication on a patient can impair the therapeutic relationship, especially if they experience side effects (2). One study on forensic prescribing found that consultants were more likely to prescribe LAIAs to patients in low and medium security and oral antipsychotics to patients in high security (2). It was postulated that since patients in high secure settings likely have a long period of supervised care ahead of them, there was less concern over treatment adherence and consequently fewer depot prescriptions (2). Indeed, some studies have shown that LAIAs can reduce relapse rates and risk of rehospitalization when compared to oral antipsychotics (other than clozapine) (5, 19, 20). This benefit may be reduced when compared to clozapine, since clozapine users usually require bi-weekly or monthly blood tests and clinic appointments to monitor for side effects, which might allow for earlier detection and management of mental deterioration to avert hospitalizations (14). The superiority of LAIAs and clozapine was confirmed in a recent observational study in 2017 from Sweden examining rehospitalization rates associated with various antipsychotics in a sample of 29,823 patients (19). The study showed that the risk of rehospitalization was lowest when using monotherapy of once-monthly LAIA paliperidone (hazard ratio [HR] 0.51), LAIA zuclopenthixol (HR 0.53), clozapine (HR 0.53), LAIA perphenazine (HR 0.58), and LAIA olanzapine (HR 0.58) (19). Furthermore, the study found that LAIA medications were associated with substantially lower risk of rehospitalization compared with equivalent oral formulations (19). Another observational study from South Korea in 2022 of over 20,000 patients found that clozapine in combination with second generation LAIAs reduced the prevalence of psychiatric admission compared to clozapine alone or LAIAs alone, and clozapine alone or augmented was superior to all other antipsychotics, regardless of formulation (21).

A recent cross-sectional study by Farrell and Brink (2020) conducted at a forensic psychiatric hospital in Western Canada, examined antipsychotic polypharmacy (i.e. prescribed two or more antipsychotics) and general prescribing patterns (1). They found a high rate of antipsychotic polypharmacy (55%) among their sample (1). Four recent studies (in the UK, Germany, and Italy) examining forensic psychiatric antipsychotic polypharmacy found rates of 12%, 50%, 22%, and 45% respectively in their samples, which highlight the variability in prescription practices from country to country, and likely province to province (2–4, 22). Farrell and Brink found that 35% of their sample was prescribed clozapine and 49% were prescribed LAIAs (1). It is estimated that 25 to 33% of people with schizophrenia are treatment resistant, and this percentage is likely higher in forensic samples, although this has not been formally investigated (11). LAIAs are variably prescribed for SSD across the globe ranging from 10% in the US, 23% in China, and 25% in Australia, to more than 30% in European countries (23). The utilization of clozapine and LAIAs at the Forensic Psychiatric Hospital in British Columbia, Canada, is encouraged by the literature, and while antipsychotic polypharmacy is not encouraged by guidelines, new research suggests it may decrease hospitalization without compromising safety (5, 6, 8, 9, 24).

It is useful to compare prescription patterns between forensic institutions and general psychiatry; yet there is almost no research available that has studied the effectiveness of pharmacological treatments in forensic populations (25). A systematic review published in 2020 found only 10 poor quality studies in this area (25). Five of the studies suggested positive effects of clozapine in reducing aggression, time to rehospitalization or reoffending, and time to discharge post-treatment, improving clinical function, and increasing crime-free time (25). However, all these studies had a high risk for bias and their evidence is considered low-grade (25). Rezansoff and colleagues showed that a medication adherence rate of >80% significantly decreased recidivism of violent and non-violent crimes in forensic patients with schizophrenia (26). They did not examine the type of antipsychotic medication or its formulation and suggested this as an area in need of further research (26).

The majority of offences leading to a forensic admission are for crimes against the person and many forensic patients have perpetrated offences covered widely in the media; as such, their return to the community can be highly contentious (27–32). Moreover, adverse outcomes following community return/discharge come with high costs for the patients, their families, and society (public safety, economic burden) (for a general discussion, see, Nicholls & Goossens, 2017; 33). Although community reintegration of forensic patients can be highly charged for the individual, their family, and society, very little is known about a key factor in discharge planning: discharge medication. Specifically, there is a need for more information about what medications forensic patients are prescribed when departing hospital on either conditional or absolute discharge, and how different medications or formulations may impact recidivism, re-hospitalization, and community functioning. Furthermore, advancing treatment and improving patient outcomes of this high-profile population could help to reduce stigma toward individuals with mental illness and people with criminal justice involvement. In this study we examined antipsychotic prescribing patterns and rehospitalization rates in a sample of patients discharged from a forensic psychiatric hospital in British Columbia, Canada.

## Methods

### Design and setting

This study is part of a larger project (the National Trajectory Project, Part 2 – Community; NTP-C; 34). NTP-C is a longitudinal, retrospective multi-site design sampling all persons found Not Criminally Responsible on Account of Mental Disorder (NCRMD) and discharged from hospitals in NS, QC, ON, MB, SK, AB and BC (Crocker, Nicholls et al., CIHR 2017-2025). Our study makes use of the BC data of this larger pan-Canadian cohort of persons found NCRMD and who were discharged from the hospital between January 1, 2010, to December 31, 2015. Follow up was until December 31, 2017. There is only one Forensic Psychiatric Hospital (FPH) in BC, Canada. The FPH is a 190-bed facility (96 high/medium secure beds and 94 low secure beds).

### Sample

Inclusion criteria were the following: 1. Any person with a Not Criminally Responsible on Account of Mental Disorder (NCRMD) verdict and hospitalized at the Forensic Psychiatric Hospital in BC; 2. who received an “official administrative discharge from the hospital”, as recorded in the archives between January 1, 2010, to December 31, 2015. “Official administrative discharge from the hospital” was defined as a conditional or absolute discharge from the FPH. Patients who absconded or died during the study period are not considered an administrative discharge. If a person had more than one release date within the five-year timeframe, the first was used and subsequent hospitalizations were included as outcomes in the data gathering system. 3. The patient had to have received an NCRMD verdict prior to being discharged from FPH during the specified time frame (rather than after). Finally (4), The patient had to have a diagnosis of a psychotic illness at the time of discharge and be treated with an antipsychotic. Psychotic illness was defined as any DSM-5 diagnosis of a psychotic illness under ‘Schizophrenia Spectrum and Other Psychotic Disorders,’ as well as bipolar disorder with psychotic features in the DSM-5’s ‘Bipolar and Related Disorders’ and major depressive disorder with psychotic features in the DSM-5’s ‘Depressive Disorders’ (35).

Patients were excluded if they were (1) an adolescent (<19 years) under the jurisdiction of the Review Board at the time of discharge. A person who was an adolescent at the time of the initial NCRMD finding could be included in the study provided they were an adult at discharge. (2) An NCRMD patient hospitalized and discharged from FPH for non-psychiatric reasons. (3) A patient for whom information regarding their prescription medication (i.e. lacking doses and types of antipsychotics) was incompletely documented within 60 days of their discharge. The initial sample size of 199 individuals who fit criteria was reduced to a final *N* = 153. A total of 46 patients were excluded due to not having a psychotic illness (12), not being prescribed an antipsychotic (10), or not having a report detailing their medications within 60 days of discharge (24).

### Procedure

Trained research assistants with a minimum of an undergraduate degree in a relevant discipline coded and entered review board and hospital file data into a bilingual computerized database to ensure standardization of data collection across study sites. A password-protected blog was maintained on our NTP-C team website to allow discussions between research assistants, project coordinators, and investigators about challenging or unusual cases (e.g., if a patient transferred between provinces). An in-depth protocol (>100 pages) was sent out to all research assistants showing the drop-down menus for each data entry. Information regarding our outcomes of interest (e.g. medications and rehospitalization) were coded up until the end of the follow up period of December 31, 2017. Ethics and institutional approvals were obtained at all relevant universities and affiliated hospitals for the full NTP-C project. Specific to this project, the University of British Columbia and BC Mental Health and Substance Use Services research committee approved the study. Following the coding of the data for NTP-C, we gathered and organized relevant variables into SPSS, and rearranged them as needed in order to analyze medications and rehospitalization information more closely.

### Data sources and variables

#### Patient Characteristics

The primary source of information was clinical files. Patients’ records were used to collect details about 3 primary categories: socio-demographic (e.g. age or sex), clinical (e.g. diagnosis), and forensic characteristics (e.g. index offense); antipsychotic medication information at discharge of index hospitalization; forensic rehospitalizations following index including date, number, and duration until the end of the follow up period.

#### Medication

The data used for this study is principally medication information provided to the last review board hearing prior to discharge from hospital. We coded type of medication, dose, and formulation. We allowed for 60 days between the reports detailing medication information to the review board and the patient’s discharge date, since many of these reports are submitted some time before the review board hearing, and many patients are not discharged from hospital immediately following a conditional or absolute discharge disposition from the review board (e.g. pending a placement/home in the community). Based on current practice at FPH, psychiatrists are unlikely to change antipsychotic medication once they have submitted their report to the review board, until the disposition is known.

#### Antipsychotic Prescriptions and Formulation Types

In determining the primary antipsychotic, if a patient was prescribed clozapine this was always considered to be the primary antipsychotic due to its superior efficacy, and it being first line in treatment-resistant schizophrenia (12, 14, 15, 36). If the patient was prescribed an injection, that was considered the primary antipsychotic as compared to oral medication due to guaranteed medication compliance with an administered injection, and because injections are generally not used as augmenting agents. If patients were prescribed multiple oral antipsychotics without an injection or clozapine, the antipsychotic prescribed at the highest dosage was considered the primary antipsychotic. The highest dosage was determined based on Stahl’s prescriber’s guide 7^th^ edition and the United Kingdom’s Prescribing Observatory For Mental Health Antipsychotic Dosage Ready Reckoner that looks at the percentage of maximum dosage according to the British National Formulary (37, 38). The subtherapeutic group included patients whose antipsychotics were prescribed below the recommended therapeutic dosage for treating psychosis according to Stahl’s prescriber’s guide 7^th^ edition and the product monograph for Nozinan (methotrimeprazine), because methotrimeprazine was not included in Stahl’s (37, 39). Previous studies have pointed out that subtherapeutic doses of antipsychotics are not entirely ineffective, and drug plasma levels are more accurate than doses due to individual patient differences in drug pharmacokinetics and metabolism (40–42). However, some studies have wondered if subtherapeutic doses may negatively affect outcomes for patients with psychosis, and because we did not have access to drug plasma levels, low antipsychotic doses below recommended levels was our best way of approximating a “subtherapeutic” group (40–42). The cutoffs for the subtherapeutic group were quetiapine 400 milligrams (mg) orally (PO), aripiprazole 15mg PO or 300mg every 4 weeks injected (IM), olanzapine 10mg PO, flupenthixol 3mg PO or 10mg every week IM, fluphenazine 1mg PO or 12.5mg every 2 weeks IM, ziprasidone 40mg PO, haloperidol 1mg PO or 10mg IM every 2 weeks, loxapine 60mg PO, methotrimeprazine 50mg PO, paliperidone 6mg PO or 39mg IM every month, risperidone 2mg PO or 12.5mg every 2 weeks, sulpiride 400mg PO or 600mg IM daily, and zuclopenthixol 20mg PO or 150mg every 2 weeks (37, 39). This resulted in the natural formation of four formulation types (oral, injection, clozapine, and subtherapeutic). Secondary or augmenting antipsychotics were any other antipsychotic in the clozapine group, any oral antipsychotic (besides clozapine) in the injection group, and lower dosed antipsychotics in the oral and subtherapeutic groups. One patient was prescribed both clozapine and an injection (risperidone), and in this case clozapine was considered the primary antipsychotic. We did not include as-needed antipsychotics in our analysis.

#### Rehospitalization

The follow up period was from discharge until December 31, 2017. Clinical records were used to record outcomes including return to hospital and the team’s reasons for any subsequent rehospitalization. Preventive and reactive forensic rehospitalizations were coded: preventive rehospitalizations occur when clinicians are concerned about an individual’s deteriorating status and admit the person to stabilize; reactive rehospitalizations occur after problems, which can include new offences. We specifically documented hospitalization rate, time to rehospitalization, and proportion of the follow up period spent in hospital. For rehospitalization analysis we removed patients who were absolutely discharged. Once absolutely discharged, patients are no longer under the supervision of the review board, and therefore cannot be recalled back to hospital unless they are certified or commit new offences. Therefore, their rehospitalization rate would be much lower than those that are conditionally discharged who can come back to hospital for many reasons, including voluntarily. Since there were only 8 patients that were absolutely discharged, statistical analysis of these compared to those conditionally discharged (N=145) would be of limited utility due to the small sample size and very low rehospitalization rate of the absolute group.

### Analyses

Data analysis was conducted using SPSS Version 25.0 for Windows. Descriptive analysis included frequencies, means, standard deviations, and ranges. We used Pearson’s chi-squared to test associations between categorical variables. For continuous variables, we used the Levene statistic to assess the normality of distribution based on the mean. Since, the sample was not normally distributed across all continuous variables, we used the Kruskal-Wallis test to assess the relationship between predictors and outcome variables. Statistical significance was determined at two-tailed *p* <.05.

## Results

### Sample characteristics

**Table 1** describes the sociodemographic, clinical, and forensic characteristics of the sample. The patients were primarily male (85.6%) and European/white (71.2%) with a mean age of 40.9 years (*SD* = 12.6). The most common diagnoses were schizophrenia (54.2%) and schizoaffective disorder (22.2%). Nearly two-thirds (62.1%) of the patients had a history of substance use disorder, usually involving stimulants, alcohol, opioids, benzodiazepines, or cannabis. While some patients had achieved abstinence prior to their admission, substance use is strictly prohibited at the forensic psychiatric hospital and so all patients were considered ‘in remission in a controlled environment’. We documented the most serious index offence for which patients were found NCRMD. The majority (80.4%) had committed offences against the person, including assault (44.4%) and murder/attempted murder/manslaughter (12.4%). The vast majority of patients were discharged from the forensic hospital with conditions (94.8%). Discharge housing ranged from highly controlled and specialized environments such as forensic transitional housing (39.9%) and tertiary psychiatric facilities (5.2%), to much less structured settings including living with family (18.3%) or living independently (13.1%).

**Table 1:** Sociodemographic, clinical, and forensic characteristics of the sample.

Characteristic	Number (%)/Mean (*SD*)/Range
	*N* = 153
Gender	
Male	131 (85.6)
Female	22 (14.4)
Age (in years)	
Mean (*SD*)	40.9 (12.6)
Range	19-79
Ethnicity	
European/white	109 (71.2)
Indigenous	21 (13.7)
Asian	13 (8.5)
African	3 (2.0)
Other/Mixed	2 (1.3)
Unknown	5 (3.3)
Primary Psychiatric Diagnosis	
Schizophrenia	83 (54.2)
Schizoaffective disorder	34 (22.2)
Unspecified psychotic disorder	19 (12.4)
Delusional disorder	6 (3.9)
Bipolar 1 disorder, manic episode with psychotic features	6 (3.9)
Substance-induced psychotic disorder	4 (2.6)
Major depressive disorder, major depressive episode with psychotic features	1 (0.7)
History of substance use disorder	
Yes	95 (62.1)
No	58 (37.9)
Most serious index offence	
Murder/attempted murder/manslaughter	19 (12.4)
Sexual offence	5 (3.3)
Assault	68 (44.4)
Other offence against the person (e.g. uttering threats/robbery)	31 (20.3)
Property offence (e.g. theft/arson/breaking and entering)	20 (13.1)
Other offence (e.g. failure to comply/public indecency)	10 (6.5)
Review board status at discharge	
Conditional discharge	145 (94.8)
Absolute discharge	8 (5.2)
Discharge Housing	
Forensic transitional housing (e.g., shared cottages, with 24-7 services)	61 (39.9)
Supervised facility (full supervision, e.g., tertiary hospital)	8 (5.2)
Supportive housing (some supervision)	14 (9.2)
Family home	28 (18.3)
Group home	16 (10.5)
Independent living	20 (13.1)
Other (shelter/jail/unknown)	6 (3.9)

### Antipsychotic prescribing practices and polypharmacy

As discussed previously, in order to be included in the study, patients had to have a psychotic diagnosis and to have been prescribed an antipsychotic. **Table 2** outlines the antipsychotics that patients were prescribed at the time of discharge. Atypical antipsychotics were chosen as the primary antipsychotic for 73.2% of patients, with clozapine, risperidone, and olanzapine being the most common at 19.0% each. The most commonly chosen primary typical antipsychotics were flupentixol and zuclopenthixol at 9.8% and 9.2%, respectively. Note that atypical antipsychotics were generally favored over typical antipsychotics as secondary agents (82.1% *vs* 17.9%, respectively). Quetiapine made up 40.3% of the secondary antipsychotics prescribed, but only 2.6% of the primary ones. In total, 84.3% of patients were prescribed an atypical antipsychotic (primary and/or secondary). Comparatively, about one-in-three (34.0%) patients were prescribed a typical antipsychotic. The most commonly prescribed were atypical antipsychotics including olanzapine at 26.8% of patients, risperidone at 22.9%, quetiapine at 20.3%, and clozapine at 19.0%, with typical antipsychotics flupentixol at 11.1% and zuclopenthixol at 9.2%.

**Table 2:** Antipsychotic medication prescribed at discharge.

Antipsychotic	Prescribing at Discharge *N* (%)			
	Primary	Secondary	Total Prescriptions	Total Patients
	*N* = 153	*N* = 67	*N* = 220	*N* = 153
Typical				
Flupentixol	15 (9.8)	2 (3.0)	17 (7.7)	17 (11.1)
Fluphenazine	3 (2.0)	0	3 (1.4)	3 (2.0)
Haloperidol	5 (3.3)	2 (3.0)	7 (3.2)	7 (4.6)
Loxapine	2 (1.3)	6 (9.0)	8 (3.6)	8 (5.2)
Methotrimeprazine	2 (1.3)	2 (3.0)	4 (1.8)	4 (2.6)
Zuclopenthixol	14 (9.2)	0	14 (6.4)	14 (9.2)
Total typical	41 (26.8)	12 (17.9)	53 (24.1)	52 (34.0)
Atypical				
Aripiprazole	5 (3.3)	6 (9.0)	11 (5.0)	11 (7.2)
Clozapine	29 (19.0)	0	29 (13.2)	29 (19.0)
Olanzapine	29 (19.0)	12 (17.9)	41 (18.6)	41 (26.8)
Paliperidone	10 (6.5)	0	10 (4.5)	10 (6.5)
Quetiapine	4 (2.6)	27 (40.3)	31 (14.1)	31 (20.3)
Risperidone	29 (19.0)	6 (9.0)	35 (15.9)	35 (22.9)
Sulpiride	2 (1.3)	4 (6.0)	6 (2.7)	6 (3.9)
Ziprasidone	4 (2.6)	0	4 (1.8)	4 (2.6)
Total atypical	112 (73.2)	55 (82.1)	167 (75.9)	129 (84.3)
Total	153	67	220	153

In our sample, 61 of the 153 patients were prescribed multiple antipsychotics (i.e., 67 secondary antipsychotics), resulting in antipsychotic polypharmacy in 39.9% of patients. The patients were divided into four formulation types based on the primary type of medication prescribed for the purpose of more in-depth analysis of medications. The oral, injection, clozapine, and subtherapeutic dose groups contained 52 (34.0%), 60 (39.2%), 29 (19.0%), and 12 (7.8%) patients, respectively.

The primary antipsychotics (**Table 2**) are described for each of the four antipsychotic groups in **Table 3**. In the oral antipsychotic group, the most common prescriptions were olanzapine (50.0%) and risperidone (19.2%). The injectable antipsychotic group was mostly prescribed risperidone (28.3%), flupentixol (23.3%), zuclopenthixol (21.7%), and paliperidone (16.7%). All of the patients in the clozapine group were prescribed clozapine. The subtherapeutic dose antipsychotic group contained a fairly even mixture of people receiving typical and atypical antipsychotics. Most (*N =* 11, 91.7%) of the 12 patients in the subtherapeutic group were prescribed oral medication, but one patient was prescribed a subtherapeutic dose of injectable zuclopenthixol (40mg injected every 2 weeks).

**Table 3:** Primary antipsychotic prescriptions at discharge by formulation type.

Antipsychotic	Primary Antipsychotic Prescriptions at Discharge by Formulation Type *N* (%)			
	Oral	Injection	Clozapine	Subtherapeutic
*N* = 153	*N* = 52	*N* = 60	*N* = 29	*N* = 12
Typical				
Flupentixol	1 (1.9)	14 (23.3)	0	0
Fluphenazine	0	3 (5.0)	0	0
Haloperidol	1 (1.9)	3 (5.0)	0	1 (8.3)
Loxapine	0	0	0	2 (16.7)
Methotrimeprazine	2 (3.8)	0	0	0
Zuclopenthixol	0	13 (21.7)	0	1 (8.3)
Atypical				
Aripiprazole	4 (7.7)	0	0	1 (8.3)
Clozapine	0	0	29 (100.0)	0
Olanzapine	26 (50.0)	0	0	3 (25.0)
Paliperidone	0	10 (16.7)	0	0
Quetiapine	2 (3.8)	0	0	2 (16.7)
Risperidone	10 (19.2)	17 (28.3)	0	2 (16.7)
Sulpiride	2 (3.8)	0	0	0
Ziprasidone	4 (7.7)	0	0	0

We further describe in **Table 4** the types of antipsychotics prescribed as secondary or augmenting agents to the primary antipsychotics (**Table 3**) as a function of the four formulation types delineated above. The polypharmacy rates for each formulation type are presented as well. The clozapine group had the highest rate of antipsychotic polypharmacy at 48.3%. Note that there are more prescribed antipsychotics in the oral and injection groups (23 and 27, respectively) than there are patients with polypharmacy (20 and 24, respectively). This is because of the 61 patients with antipsychotic polypharmacy, 5 of those were prescribed a third antipsychotic, and of those five patients, one was prescribed a fourth antipsychotic. Quetiapine was chosen as the secondary antipsychotic at a rate of 47.8% in the oral group, 44.4% in the injection group, and 100% in the subtherapeutic group, but only 7.1% in the clozapine group. Furthermore, aripiprazole or sulpiride were chosen to augment clozapine at a rate of 57.1%, but only 4.3% in the oral group, 3.7% in the injection group, and never in the subtherapeutic group. Another common secondary antipsychotic was olanzapine at 8.7% in the oral group, 33.3% in the injection group, and 7.1% in the clozapine group.

**Table 4:** Secondary antipsychotic prescriptions at discharge by formulation type.

Antipsychotic	Oral	Injection	Clozapine	Subtherapeutic
*N* = 67	*N* = 52	*N* = 60	*N* = 29	*N* = 12
	Number (% of *N*)			
Polypharmacy	20 (38.5)	24 (40.0)	14 (48.3)	3 (25.0)
	Number (% of total)			
Typical				
Flupentixol	1 (4.3)	1 (3.7)	0	0
Haloperidol	1 (4.3)	1 (3.7)	0	0
Loxapine	4 (17.4)	1 (3.7)	1 (7.1)	0
Methotrimeprazine	1 (4.3)	0	1 (7.1)	0
Atypical				
Aripiprazole	0	1 (3.7)	5 (35.7)	0
Olanzapine	2 (8.7)	9 (33.3)	1 (7.1)	0
Quetiapine	11 (47.8)	12 (44.4)	1 (7.1)	3 (100.0)
Risperidone	2 (8.7)	2 (7.4)	2 (14.3)	0
Sulpiride	1 (4.3)	0	3 (21.4)	0
Total	23	27	14	3

### Antipsychotic formulation types and sociodemographic associations

We examined the relationship between sociodemographic, clinical, and forensic characteristics and the four antipsychotic formulation types based on the primary antipsychotic prescribed (**Table 5**). In many cases, variables had to be aggregated for the chi-squared statistical test to be valid, and in some cases the test was never valid (gender, ethnicity, and review board status at discharge). The only significant association found was between primary psychiatric diagnosis and the antipsychotic groups (*χ²* = 19.77; *p* = .003). 33.3% of patients in the subtherapeutic group had a diagnosis of schizophrenia or schizoaffective disorder, while 71.2%, 81.7% and 93.1% of patients in the oral, injection, and clozapine groups respectively had one of these diagnoses.

**Table 5:** Associations between discharge antipsychotic formulation types and sociodemographic, clinical, and forensic characteristics.

Characteristics	Oral	Injection	Clozapine	Subtherapeutic	Stat. test	p-value
*N* = 153	*N* = 52	*N* = 60	*N* = 29	*N* = 12		
	Number (%)					
Gender					Not valid	——
Male	45 (86.5)	49 (81.7)	25 (86.2)	12 (100.0)		
Female	7 (13.5)	11 (18.3)	4 (13.8)	0		
Age					*H* = 2.39	.495
Mean (SD)	40.9 (12.8)	42.3 (12.2)	39.2 (11.3)	38.8 (17.0)		
Range	19-72	21-79	25-63	20-72		
Ethnicity					Not Valid	——
European/white	40 (76.9)	45 (75.0)	18 (62.1)	6 (50.0)		
Indigenous	5 (9.6)	7 (11.7)	4 (13.8)	5 (41.7)		
Other	7 (13.5)	8 (13.3)	7 (24.1)	1 (8.3)		
Primary Psychiatric Diagnosis					*χ²* = 19.77	.003
Schizophrenia	26 (50.0)	34 (56.7)	21 (72.4)	2 (16.7)		
Schizoaffective disorder	11 (21.2)	15 (25.0)	6 (20.7)	2 (16.7)		
Other psychotic illness	15 (28.8)	11 (18.3)	2 (6.9)	8 (66.7)		
Hx of substance use disorder					*χ²* = 6.76	.080
Yes	39 (75.0)	35 (58.3)	16 (55.2)	5 (41.7)		
No	13 (25.0)	25 (41.7)	13 (44.8)	7 (58.3)		
Most serious index offence					*χ²* = 2.04	.564
Offence against the person	41 (78.8)	47 (78.3)	26 (89.7)	9 (75.0)		
Other offence	11 (21.2)	13 (21.7)	3 (10.3)	3 (25.0)		
Review board status at D/C					Not Valid	——
Absolute discharge	3 (5.8)	4 (6.7)	0 (0.0)	1 (8.3)		
Conditional discharge	49 (94.2)	56 (93.3)	29 (100.0)	11 (91.7)		
Discharge Housing					*χ²* = 1.40	.706
Staffed housing	30 (57.7)	29 (48.3)	17 (58.6)	7 (58.3)		
Other housing (e.g. family)	22 (42.3)	31 (51.7)	12 (41.4)	5 (41.7)		

### Antipsychotic formulation types and rehospitalization

We describe the forensic rehospitalization rates of the entire sample in **Table 6**. More than half (*N =* 81, 52.9%) of the patients had at least one rehospitalization in the follow-up period. It is evident that there is wide variation in the sample, as reflected in large standard deviations. This is due to patients ranging from spending none of their time to nearly the entire (97.7%) follow up period rehospitalized. Once patients who had received absolute discharges were excluded, the remaining 145 patients who were conditionally discharged experienced on average 1.3 rehospitalizations (*SD* = 1.7). These patients spent 386.8 days rehospitalized (*SD* = 659.3) and spent 17.3% of the follow up period in hospital (*SD* = 28.2).

**Table 6:** Forensic rehospitalization rates of the sample.

Metric	Entire Sample	Excluding Absolute Discharges
	*N* = 153	*N* = 145
	Number (%)	
Any rehospitalization		
Yes	81 (52.9)	80 (55.2)
No	72 (47.1)	65 (44.8)
	Mean (SD)	
Days between discharge and end of follow up period	2107.2 (680.9)	2117.1 (675.3)
Range	741-2909	783-2909
Days to first rehospitalization*	386.8 (442.1)	390.5 (443.7)
Range	1-2529	1-2529
Number of rehospitalizations	1.2 (1.7)	1.3 (1.7)
Range	0-8	0-8
Total days rehospitalized	366.6 (647.4)	386.8 (659.3)
Range	0-2584	0-2584
Proportion of follow up period rehospitalized (%)	16.4 (27.7)	17.3 (28.2)
Range	0-97.7	0-97.7
Average length of rehospitalization(s) (days)	204.6 (414.9)	215.8 (423.4)
Range	0-2584	0-2584

*Days to first rehospitalization only includes those patients who were rehospitalized (*N* = 81 or *N* = 80).

An analysis of associations between rehospitalization rates and each of the antipsychotic formulation types is presented in **Table 7**. More than half of the people in the oral group (*N =* 28, 57.1%) and the injection group (*N =* 33, 58.9%) were rehospitalized, compared to just under half (*N =* 14, 48.3%) of the people in the clozapine group. In the subtherapeutic group, five (45.5%) patients were rehospitalized. On average, the patients in the clozapine group were rehospitalized for 10.7% (*SD* = 23.5) of the follow up period. In comparison, people in the injection group (20.5%, *SD* = 29.8) and people in the oral group (21.0%, *SD* = 30.5) were rehospitalized for one-fifth of the follow-up period. In the subtherapeutic group, patients were rehospitalized for 2.3% (*SD* = 4.5) of the follow up period. Similarly, the number of rehospitalizations was 0.7 (*SD* = 0.9) for the clozapine group, compared to 1.5 (*SD* = 1.8) in the injection group, 1.5 (*SD* = 1.9) in the oral group, and 0.6 (*SD* = 0.9) in the subtherapeutic group.

## Discussion

There are very few studies that examine the relationships between different antipsychotic formulation types and rehospitalization in forensic psychiatric samples. We found that more than half of conditionally discharged patients had a rehospitalization during the follow up period, although the time patients spent in hospital varied significantly between patients. Many of these rehospitalizations were voluntary or preventive, often due to patient anxiety or deterioration of mental state, rather than new offences. Overall, we did not find a significant difference between the four groups regarding rehospitalization rates. Although the number of rehospitalizations, the total days rehospitalized, and the proportion of the follow up period spent in hospital all favored clozapine compared to injection and oral antipsychotics (but not subtherapeutic dose), the large standard deviations and relatively small sample size did not provide enough power to achieve significance. However, on average the clozapine group spent about half of the time rehospitalized (~10% *vs* ~20%) compared to the oral and injection groups. While clozapine’s superior efficacy could be the reason for this, a potential confounder is the increased monitoring and follow up that clozapine requires, which could on its own improve outcomes (43). Clozapine’s potential benefit may represent an important trend that is also seen in general psychiatric samples, but more research in forensic settings is needed (14, 19, 21).

A somewhat surprising finding was that LAIAs did not provide any advantage in terms of rehospitalization rates compared to prescribing only oral antipsychotics. A recent study showed that prescribing LAIAs at discharge in forensic psychiatric patients was associated with better treatment adherence compared to those prescribed only oral antipsychotics, but also found no difference in rehospitalization rates (44). There could be many reasons for this result, including clinical treatment selection, where patients with more severe psychopathology that have previously failed oral antipsychotics or are known to be non-adherent to medications, are prescribed injectable antipsychotics. However, general psychiatry would also have this same selection bias, where the benefits of LAIAs on reducing rehospitalization rates have been demonstrated (5, 19, 20). These results highlight that different findings may emerge in forensic psychiatric patients, in contrast to general psychiatric patients.

When we compared our antipsychotic groups regarding clinical and demographic variables, we found a significant difference with regard to diagnosis. At least 70% of patients in the oral, injection, and clozapine groups had a diagnosis of schizophrenia or schizoaffective disorder, while only a third of patients in the subtherapeutic group had these diagnoses. This is as expected because from a clinical point of view, psychosis associated with schizophrenia and schizoaffective disorder would be considered more severe than substance-induced, unspecified or mood related psychotic disorders, warranting higher doses of antipsychotic medication. In fact, a recent study published in the Lancet, noted that “in multi-episode schizophrenia, antipsychotic doses should probably not be reduced below the standard dose range recommended for acute stabilization, because reducing the dose further is associated with an increased risk of both relapse and all-cause discontinuation” (45). However, this may not apply to psychoses other than schizophrenia and schizoaffective disorder, and interestingly, the subtherapeutic group that was much less likely to have those disorders, also had numerically lower rates of rehospitalization than the other groups (although not significant). Indeed, brief psychotic episodes, which may be due to substance use, part of a mood disorder, or occur for unknown reasons, do not have their own evidence-based treatment guidelines, and can represent a diagnostic and treatment conundrum (46).

This study also described the demographics and antipsychotic prescribing practices in a Canadian forensic psychiatric sample. Our sample was similar to previous studies conducted in forensic samples and specifically in Canadian samples, with the majority of the patients being male, Caucasian, middle aged, and having a schizophrenia spectrum diagnosis (1, 28). Interestingly, females represented 14.4% of the sample, which is higher than previous estimates of 6 to 10% and supports the hypothesis that female involvement in forensic mental health services may be on the rise (28, 47). We also found that many patients (54.2%) were discharged to some type of supervised or supportive housing, which is supported by prior research that has found improved outcomes (i.e., decreased recidivism) for this housing type (48).

In terms of antipsychotic prescriptions (**Tables 2**–**4**, **7**), we elected to divide them into primary and secondary antipsychotics, based on dosage and/or formulation. This provides information regarding which antipsychotic is considered the one doing the majority of treatment, and which one is augmenting it. As in other studies, a significant majority of the sample was prescribed atypical antipsychotics (84.3%), the main medications including clozapine, olanzapine, risperidone, and quetiapine (1, 2). This is likely due to concerns regarding the extrapyramidal side effects of typical antipsychotics. Interestingly, quetiapine represented 40.3% of the augmenting or secondary antipsychotic prescriptions, but only 2.6% of the primary ones. Many of these patients were prescribed a low dose of quetiapine at nighttime, which in our experience is a common off-label use of this medication, for sleep and/or mood instability. Quetiapine is a first line treatment in Canada for bipolar depression and second line treatment for unipolar depression (49, 50). Around 40% of patients were prescribed an injectable antipsychotic, with just over half of these being typical antipsychotics, again in line with previous studies (1, 2). The polypharmacy rate of 39.9% was lower than a previous study at the same hospital, and there were no significant differences between the clozapine, injection, and other groups (1). This is likely due to the previous study being a cross-section of admitted patients, while this study examined discharge medications; often patients experience transient polypharmacy during admission while they are switching from one antipsychotic to another.

**Table 7:** Associations between antipsychotic formulation types at discharge from index hospitalization and aspects of rehospitalizations.

Rehospitalization characteristics	Oral	Injection	Clozapine	Subtherapeutic	Statistic	*p-*value
	*N* = 49	*N* = 56	*N* = 29	*N* = 11		
	Number (%)					
Any rehospitalization					*χ²* = 1.37	.712
Yes	28 (57.1)	33 (58.9)	14 (48.3)	5 (45.5)		
No	21 (42.9)	23 (41.1)	15 (51.7)	6 (54.5)		
	Mean (*SD*)					
Days between discharge and end of follow up period	2244.1 (640.1)	2035.6 (722.5)	2094.9 (658.5)	2024.2 (623.7)	*H* = 3.29	.349
Days to first rehospitalization*	320.8 (365.5)	450.2 (530.8)	431.4 (422.2)	272.4 (256.3)	*H* = 2.11	.550
Number of rehospitalizations	1.5 (1.9)	1.5 (1.8)	0.7 (0.9)	0.6 (0.9)	*H* = 4.48	.214
Total days rehospitalized	518.6 (770.2)	394.5 (622.6)	274.9 (604.8)	55.2 (110.2)	*H* = 4.81	.186
Proportion of follow up period rehospitalized (% days)	21.0 (30.5)	20.5 (29.8)	10.7 (23.5)	2.3 (4.5)	*H* = 5.44	.142
Average length of rehospitalization(s) (days)	249.5 (419.4)	219.9 (400.4)	219.4 (537.6)	35.6 (69.1)	*H* = 3.61	.307

Days to first rehospitalization only includes those patients who were rehospitalized (*N* = 80).

Clozapine was prescribed for 19% of patients in our sample, which is lower than the accepted prevalence for the treatment of people with resistant schizophrenia, and lower than the amount prescribed to patients in prior forensic studies (1, 2). Although we did not confirm treatment resistance in our sample, it is generally accepted that forensic patients are more treatment resistant than general psychiatry patients, of which approximately one third of those with schizophrenia are treatment resistant (4). We expect that clozapine was prescribed to more than 19% of our patient sample, but some patients could not tolerate the medication due to side effects such as myocarditis or orthostatic hypotension. Another explanation would be that clozapine is being underutilized at our hospital, although further research is needed to determine the lower percentage of prescriptions. We noted that more than half of the patients with clozapine polypharmacy had been augmented with aripiprazole or sulpiride, whereas these medications accounted for less than 5% of the augmenting medications in the oral, injection, and subtherapeutic groups. Although the overall evidence for clozapine augmentation is poor, there are some short-term studies that have suggested a particular benefit of clozapine augmentation with aripiprazole followed by sulpiride (51–53).

While we have described informative trends in this population, it would be beneficial to compare this with other forensic settings in Canada. Given the larger sample size of a study that involves several Canadian provinces, we may have the power to better compare antipsychotic effectiveness and show a trend that would likely favor clozapine regarding rehospitalization, as it does in general psychiatry (14, 19, 21). Further research in this area will address substantial gaps in the literature and inform the treatment of our country’s most challenging psychiatric patients.

### Strengths and limitations

The discharge prescription was not included in the NTP-community study file review; however, we coded in detail the medications the patients were taking at the time of their last review board hearing prior to discharge. Since the last review board hearing is typically only days to weeks before discharge from hospital, and since the patient is felt to be stable enough for discharge at the last review board hearing, it is unlikely in our experience that there would be any significant changes made to medications between then and the discharge date. Therefore, we feel that this is an acceptable estimate of the discharge medications if medication information is provided within 60 days of the discharge date. Regarding medications, many patients were also prescribed other psychotropic drugs such as mood stabilizers or antidepressants, and given the small sample size, it cannot be assumed that these additional psychotropics were randomly distributed in this cohort. While we elected to focus on antipsychotics in this study, additional psychotropics in people with a psychotic illness could alter outcomes, such as re-hospitalization. Future studies with larger sample sizes could explore expanded medication comparisons. In terms of rehospitalization, the exact medications at re-admission or medication changes made during repeat hospitalizations were not coded in the chart reviews. Therefore, we do not know if there had been significant medication changes while the patient was out of hospital, which may have had a role in their relapse and re-hospitalization. However, after a patient has been stabilized at a forensic hospital and discharged, in our experience, psychiatrists tend to be reticent to make changes to the patient’s antipsychotic medication regime in the community, due to concerns around relapse and recidivism. In addition, the vast majority of patients were conditionally discharged, and therefore other psychiatrists not connected to hospital-based care would be unlikely to make significant changes to medications unless necessary. Therefore, we believe the re-hospitalization data still provide valuable correlate information to psychiatrists regarding antipsychotic efficacy in a forensic sample and provides a stepping-stone for further research.

## Conclusions

Overall, this study provides new and valuable information regarding antipsychotic prescriptions and rehospitalization in a representative forensic psychiatric sample. We found that antipsychotic prescribing practices in our population, such as a preponderance of atypical antipsychotics, antipsychotic polypharmacy rates, and augmentation of clozapine with aripiprazole or sulpiride, were generally in line with previous research. Clozapine may be underutilized in our sample, although further research is needed for clarification. Regarding rehospitalization, people prescribed clozapine had numerically lower rates of rehospitalization than patients on oral and injection formulation types, but it was not statistically significant. Factors that may have affected statistical significance were the size of our sample and the sample’s non-normal distribution. Future research with a larger sample size could potentially demonstrate the benefits of clozapine regarding rehospitalization as in general psychiatry. Previous systematic reviews have highlighted the need for high quality studies examining the effectiveness of pharmacological treatment in forensic psychiatry; better treatments for forensic patients will help reduce the significant stigma and institutionalization that these patients face.

## Data Availability

The original contributions presented in the study are included in the article/supplementary material. Further inquiries can be directed to the corresponding author.
